# Anti-Arthritic Activity of *Schistosoma mansoni* and *Trichinella spiralis* Derived-Antigens in Adjuvant Arthritis in Rats: Role of FOXP3^+^ Treg Cells

**DOI:** 10.1371/journal.pone.0165916

**Published:** 2016-11-01

**Authors:** Maha M. Eissa, Dalia K. Mostafa, Amany A. Ghazy, Mervat Z. El azzouni, Laila M. Boulos, Layla K. Younis

**Affiliations:** 1 Department of Medical Parasitology, Faculty of Medicine, Alexandria University, Alexandria, Egypt; 2 Department of Clinical Pharmacology, Faculty of Medicine, Alexandria University, Alexandria, Egypt; 3 Department of Medical microbiology and Immunology, Faculty of Medicine, Kafrelsheikh University, Kafrelsheikh, Egypt; 4 Department of Pathology, Faculty of Medicine, Alexandria University, Alexandria, Egypt; Penn State University, UNITED STATES

## Abstract

A growing body of evidence supports the concept of helminths therapy in a variety of autoimmune diseases. Here, we aimed to investigate the protective effects of autoclaved *Schistosoma mansoni* antigen (ASMA) and *Trichinella spiralis* antigen (ATSA) on the clinical and immunopathological features of rheumatoid arthritis (RA). Adjuvant arthritis was induced by subcutaneous and intradermal injections of complete Freund’s adjuvant into the plantar surface of the right hind paw and the root of the tail, respectively. Rats were randomly assigned to serve as normal control, untreated arthritis, ASMA or ATSA-treated arthritis groups. Antigens were given by intradermal injection in two doses, two weeks apart. The development, progression of arthritic features, and the impact on animals’ gait and body weight were followed up for 4 weeks. The associated changes in serum cytokines (IL-17, IFN-γ and IL-10), joints’ histopathology and immunohistochemistry of Foxp3^+^ T regulatory cells (Tregs) were evaluated at the end of the study. Treatment with either ASMA or ATSA attenuated the progression of clinical features of polyarthritis, improved gait and body weight gain, reduced the elevated serum IL-17 and further increased both IFN-γ and IL-10. Histopathologically, this was associated with a remarkable regression of paws’ inflammation that was limited only to the subcutaneous tissue, and a significant increase in the number of Foxp 3+ cells versus the untreated arthritis group. In conclusion, both *Schistosoma mansoni* and *Trichinella spiralis* derived antigens exerted protective effect against adjuvant arthritis with better effect achieved by ASMA treatment. This anti-arthritic activity is attributed to upregulation of the Foxp3^+^ Tregs, with subsequent favorable modulation of both pro- and anti-inflammatory cytokines. The use of autoclaved parasitic antigens excludes the deleterious effects of imposing helminthic infection by using live parasites, which may pave the way to a new therapeutic modality in treating RA.

## Introduction

Rheumatoid arthritis (RA) is a chronic, systemic, immune mediated inflammatory disease associated with decreased life expectancy and impaired quality of life. This devastating disease is characterized by chronic inflammation and synovial hyperplasia leading to destruction of cartilage and bone with its consequence of permanent disability [1].

A better understanding of the pathophysiology of RA has led to significant improvements in the various lines of treatment i.e. disease modifying anti-rheumatic drugs (DMARDs) and biologic therapy, aiming to achieve a remission and prevent further damage of the joints without causing side effects. However, limited effectiveness and adverse effects of the current therapies highlight the urgent need for alternative treatment [1].

A large body of epidemiological data supports the hypothesis that infection with helminthes might provide some protection against autoimmune diseases [2–6]. The potential of helminthic infection to strongly influence the immune system and to enable protective pathways has begun to be realized in various immune disorders, including diabetes mellitus, ulcerative colitis, Crohn’s disease as well as multiple sclerosis [7–9]. The consideration of the impact of infection with helminthes on arthritic disease is limited, but available data support the general concept of “helminth therapy “[10]. The general consensus is based on the reciprocity in immune regulation where Th2 cell derived mediators evoked by helminthic infection inhibit the activity of Th1 cells. Therefore, hypothetically, infection with helminthic parasites could be used to treat disease driven by Th1 cells like RA [10].

Specific helminthic species that can be safely used to treat specific human diseases in appropriate candidate patients should therefore be identified. However, it remains the art of convincing a patient to get infected by a parasite to ameliorate another disease. Here arises the concept of using helminthic derived molecules or antigens instead [11–13] *Schistosoma mansoni* (*S*. *mansoni*) is one of the few helminthes that has been reported to have an effect in arthritic disease. Infection with this parasite was shown to reduce the severity of collagen induced arthritis in mice through systemic and local suppression of pro-inflammatory mediators, suggesting their substantial benefit as therapeutic agents against RA [14]. Nevertheless, the potential drawbacks to their use, due to the disease they cause, offset the proposed benefit. Therefore, investigating the assumption that *S*. *mansoni* derived antigen(s) would have the same ameliorative activity in RA is not only a basic interest but also may have important clinical application. On the other hand, accumulating experimental evidence called attention to *Trichinella spiralis* infection as a promising therapeutic strategy in various allergic and autoimmune diseases including Type 1 diabetes [15] air way disease, inflammatory bowel disease and autoimmune encephalitis [16–18]. However, its role in arthritis has not yet been investigated. Therefore, this study was conducted to investigate the possible modulatory effect of *S*. *mansoni* and *T*. *spiralis* antigens on the course and severity of RA like changes in a rat model of adjuvant arthritis (AA). It also aimed to uncover the role of these helminthic antigens on the skewed immune response in this model of arthritis. We used complete Freund’s adjuvant (CFA) to induce arthritic immunopathological disease that displays many of the pathological features of RA. This arthritis model has been widely used for preclinical testing of numerous anti-arthritic agents which are either under preclinical or clinical investigation, or are currently used as therapeutics in this disease. The reliable onset and the progression of robust, easily measurable poly-articular inflammation offered an opportunity to study the immune modulatory effect of the investigated parasitic antigens on RA like changes [19].

## Materials and Methods

### Ethics statement

Animal studies reported are in compliance with the ARRIVE guidelines [20]. The experimental protocol was approved by the Ethics Committee of the Faculty of Medicine, Alexandria University (permit No 0302865). Thiopental Sodium was used for anesthesia and all efforts were made to minimize animal suffering, and animal care was according to the NIH Guide for care and use of laboratory animals.

### Preparation of parasitic antigens

Parasitic antigens were prepared from the infective stages of two different parasites, *Schistosoma mansoni* (cercariae) and *Trichinells spiralis* (larvae). The life cycles of both parasites were maintained at the laboratory of Medical Parasitology Department, Faculty of Medicine, Alexandria University, Egypt. For *S*. *mansoni*, it was propagated in *Biomphalaria alexandrina* mollusks as the intermediate host [21], while male CD1 albino mice were used for the mammalian stages [22].

For *T*. *spiralis*, it was maintained by serial passages in male CD1 mice (Wasson *et al*., 1988). Parasitic antigens were prepared by concentrating the freshly liberated cercariae of *S*. *mansoni* from infected snails, by gravity sedimentation at 4°C [23] and by collecting the larvae of *T*. *spiralis* from infected mice by the digestion method and their concentration by gravity sedimentation [24]. Both parasitic stages were autoclaved under pressure of 15 Ib at 121°C for 15 minutes [25]. Autoclaved *Schistosoma mansoni* antigen (ASMA) and autoclaved *Trichinella spiralis* antigen (ATSA) were stored at -20°C until being lyophilized. The protein content of both antigens were estimated according to the method of Lowry et al 1951 [26].

### Experimental animals and design

A total of 34 adult female Wistar rats (12–16 weeks old) weighing 180-200g, purchased from the animal house of Medical Research Institute, Alexandria University, Alexandria, Egypt, were used to perform the experiment. Rats were maintained under standard laboratory conditions and 12:12 light/dark cycles with free access to food and water ad-libitum. They were allowed to acclimatize for one week to the housing conditions in the animal house of Medical Parasitology Department, before conduction of the experiments.

Rats were randomly divided into four groups of 8 rats each: normal control, untreated arthritis group, ASMA-treated, and ATSA-treated arthritis groups. Adjuvant arthritis (AA) was induced in all rats except for those of normal control by subcutaneous (SC) injection of 0.1 ml CFA (Sigma-Aldrich, USA) into the plantar surface of the right hind paw. Another booster intra-dermal injection of 0.1 ml was given into the root of the tail on the same and on the following day [27]. Normal control rats received SC injection of an equal volume of saline instead in the same anatomical site. Once the early symptoms of local arthritis appeared on the day after the induction of arthritis, either ASMA or ATSA antigens were given to rats in their corresponding groups. Parasitic antigens were given to arthritic rats by intradermal injection of 5 ug/kg of ASMA [28, 29] or 70 mg/kg of ATSA [30] into the skin over the sternal region. Repeated dosing of both antigens was given two weeks later, while rats in both the normal control and arthritis groups received equal volume of saline instead. Paracetamol in a dose of 50 mg/kg/day was administered to all animals to alleviate the expected severe pain associated with the acute inflammation during the first week after induction of arthritis [31]. The protective effect of the parasitic antigens against AA was evaluated in terms of clinical assessment of the progression of arthritis and change in the body weight, biochemical estimates of inflammatory cytokines and histopathological examination of the inflamed joints. Humane endpoints used during the study were: rapid weight loss of >20% of body weight that does not begin to reverse within 5 days, poor physical appearance in the form of scruffy fur and hunched body, severe paw ulceration with no signs of healing within 3 days, sledging and impaired ambulation which prevented animals from reaching food or water [31]. Two rats reached one of the humane endpoints, and they were euthanized by an over dose of thiopental sodium. They were further substituted to keep the constant number of the animal groups.

### Experimental procedures

#### Clinical assessment of progression of arthritis

Animals were assessed for progression of AA and change in body weight at selected time points throughout the study period. Clinical signs of inflammation were evaluated on day zero and day one, then every three days till the 28^th^ day after CFA injection. Paws were examined and graded for the severity of erythema and swelling using a 5-point scale: 0 = no signs of inflammation, 1 = swelling and erythema of the digit, 2 = moderate swelling and erythema, 3 = severe swelling and erythema involving the whole area down to the ankle and 4 = severe swelling, erythema, gross deformity and disability to use the limb. The sum of scores for the 4 paws was used to calculate an arthritic score as a semi quantitative assessment of polyarthritis severity; a well-established, widely used scoring system. The maximum arthritic score per rat was set at 16 (4 points×4 paws) [27].

Paw size was determined by measuring the mediolateral and the superior inferior/diameters, using Vernier caliper which is accurate to 0.02 mm [14]. Pre-injection values for paw size were measured just prior to CFA injection for each rat and were used as a baseline. The severity of arthritis pain was measured using a modified gait scoring system based on the walking pattern of arthritic rats. In a quiet, dimmed room, each rat was placed on an open bench that enabled the animal to walk freely. The severity of disturbances of walking was graded as a semi quantitative parameter from 0 to 3 where 0: normal; rat runs and walks normally, 1: mild disability; rat runs and walks with difficulty, 2: rat walks with difficulty due to intermittent loading of inflamed joint, and 3: rat stands on only three paws i.e. total joint immobility [32]. At the beginning of the study, baseline body weight was also recorded and then it was followed weekly. The percentage of increase or decrease in the body weight relative to the starting weight was calculated for each rat.

**Fig 1:**
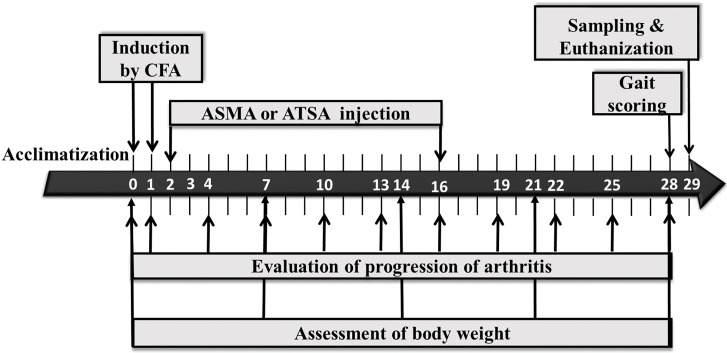
Illustration of the time line for induction of arthritis, antigen administration and experimental procedures. CFA, complete Freund’s adjuvant; ASMA, autoclaved *Schistosoma mansoni* antigen; ATSA, autoclaved *Trichinella spiralis* antigen.

The experimental schedule of antigens administration and experimental procedures are shown in Fig 1.

After 28 days of induction of arthritis, animals were anaesthetized by thiopental sodium for collection of blood for biochemical analysis, then they were further euthanized by an extra-dose of thiopental sodium anesthesia (150 mg/kg, i.p.) for dissection of paws, and all efforts were made to minimize animal suffering. The collected tissues were prepared for histopathological examination.

#### Biochemical analysis

Blood was collected from the abdominal aorta, and centrifuged at 1000 g for 10 minutes for separation of serum. Serum samples were stored at −80°C for further estimation of inflammatory cytokines. IL-17, IL-10 and IFN-γ were determined using rat ELISA kits (eBioscience, Vienna, Austria) according to the manufacturer^’^s protocol. The colour change was measured spectrophotometrically at a wavelength of 450 nm. The concentrations were calculated based on standards and expressed in pg. ml.^-1^.

#### Histopathological and immunohistochemical examination

Right and left joints of hind limbs and fore limbs were dissected and isolated for histopathological examination. Isolated tissues specimens were decalcified with diluted nitric acid, fixed in 10% neutral buffered formalin and processed for routine paraffin block preparation. Multiple representative sections (3um thick) were cut and stained with hematoxylin and eosin (H&E) for microscopic examination [33]. The extent of joint inflammation and destruction of bone and cartilage was determined using a semi-quantitiative modified composite graded scale: grade 0, no signs of inflammation; grade 1, mild inflammation with hyperplasia of the synovial lining and minor cartilage damage; grades 2 through 4, increasing degrees of inflammatory cell infiltrate and destruction of bone and cartilage [34]. Immunohistochemical identification of forkhead box (foxP3)^+^ T regulatory (Treg) cells in joint tissues was performed using rat anti-foxp3 antibody, clone 150D/E4 (eBioscience, Vienna, Austria). The detection kit used was ultravision detection system anti-polyvalent horseradish peroxidase/ diaminobenzidine tetra-hydrochloride (HRP/DAB). Joint tissue sections were deparaffinised, hydrated, subjected to microwave antigen retrieval in citrate buffer for 15 minutes and blocked for endogenous peroxidase, then they were exposed to the primary antibody (antifoxp3) for three hours at a dilution of 1:50. Biotinylated goat antipolyvalent was applied for one hour, then streptavidin biotin was applied for 20 minutes. The chromogen used was DAB. Tonsils are used as a positive control for each run, while negative control was used by omission of the primary antibody. A minimum of 3 high power fields (HPFs x400) were counted for each section and positive cells were expressed as the mean number of positive cells /HPF.

### Statistical analysis

All parametric data were analyzed by one-way analysis of variance (ANOVA) followed by the Least Significance Difference (LSD) criterion for multiple comparison. For the nonparametric data as those of arthritic and gait scores, Kruskal-Wallis one way ANOVA was used followed by the Dunn’s multiple comparison test. Analysis was performed by an investigator who was blind to the key code of the experimental animals and was done using Statistical package: MATLAB Statistical toolbox (Matrices Laboratory software-MathWorks ^®^, Model No, R2008b; full version No, 7.7.0.471-R2008b, The Math Works Inc., Natick, Massachusetts, USA) and data were expressed as means ± S.E.M or medians for parametric and nonparametric data, respectively. Statistical significance was set at P < 0.05.

## Results

### Arthritis induction and progression

**Fig 2:**
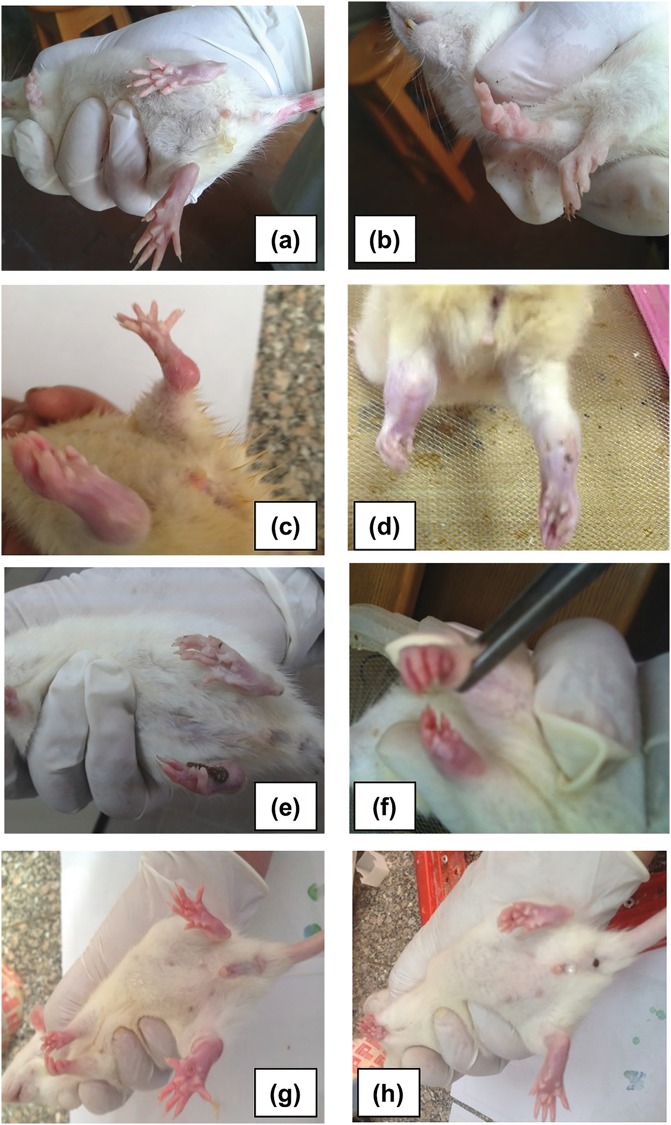
Representative photograph showing clinical signs of inflammation in rats’ joints examined on day 22 after CFA injection. ***(a*, *b)***, normal control rats, ***(c-f)*** untreated arthritic rats showing severe swelling and redness in both hind paws involving the metatarsal joints and extending to the ankle joint in ***(c)*** and ***(d)***. Note the severe erosion and amputated toes in ***(e)*,** while less severe swelling was observed in the forepaws in ***(f)*.** Rats from the ASMA ***(g)*** and ATSA ***(h)-***treated arthritis groups showing less intense inflammatory signs in the right hind paw and almost ameliorated inflammation in the other paws**.** CFA, complete Freund’s adjuvant; ASMA, autoclaved *Schistosoma mansoni* antigen and ATSA, autoclaved *Trichinella spiralis* antigen.

**Fig 3:**
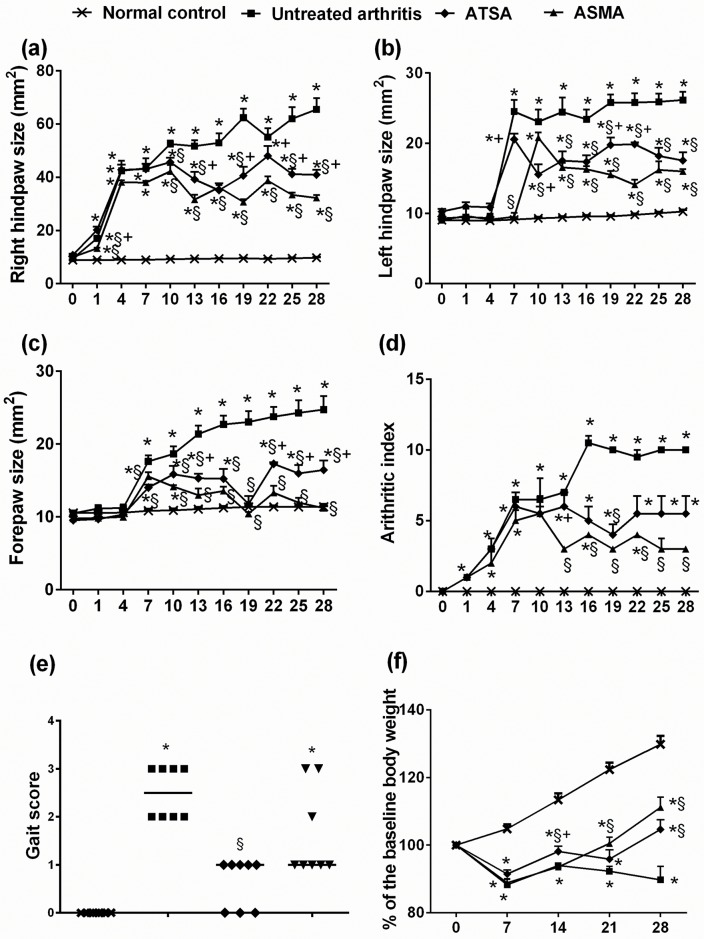
Effect of treatment with ASMA or ATSA on CFA-induced changes in the right paws’ ***(a)***, left paws’ ***(b)*** and forepaws’ ***(c)*** diameters. An overall arthritic score considering the degree and the number of affected joints is presented in ***(d)***, while a scatter plot for the gait score is presented in ***(e)***. ***(f)*** shows the time course of the encountered changes in the body weight as percentage from the baseline body weight. Data are expressed as means ± S.E.M. in ***(a)***, ***(b)***, ***(c)*** and ***(f)***, as medians ± interquartile range in ***(d)*** and as median values in ***(e)***. *p< 0.05 versus normal control group, **§***p*< 0.05 versus untreated arthritis group and +*p*<0.05 versus ASMA-treated arthritis group. CFA, complete Freund’s adjuvant; ASMA, autoclaved *Schistosoma mansoni* antigen and ATSA, autoclaved *Trichinella spiralis* antigen.

All treated and untreated-CFA injected rats started to show signs of inflammation in the injected right paw few hours after the injection that were clearly manifested after 24 hours and remained thereafter**.** This was detected by a significant increase in joint diameters (*p* = 0.0000), redness, and hotness that were progressively increasing with time (Figs 2c, 2d and 3a). It is noteworthy that some rats in the untreated arthritis group exhibited severe inflammatory changes to the extent of erosion and sloughing of the skin with the exposure of the underlying tendons. Spontaneous amputation of the toes was observed in two rats (Fig 2e). After one week, a significant increase of the size of the contralateral paw was detected (*p* = 0.0000) together with a noticeable redness in the untreated arthritic rats (Figs 2c, 2d and 3b). The forepaws exhibited a similar but less intense inflammatory signs (Figs 2f and 3c) leading to a significant increase (*p*<0.001) in the overall arthritic score versus normal control rats (Fig 3d).

Concurrent treatment with either ASMA or ATSA significantly attenuated the progression of swelling in all joints (Fig 2g and 2h) throughout the whole course (*p<*0.05), though it was fluctuating (Fig 3a–3c). A significant difference in joint diameters was also observed between the ASMA and ASTA treated rats (*p<*0.05), in favour of a better response with ASMA treatment. However, this difference was not consistent throughout the days of assessment. The improvement in other signs of inflammation together with decreased joint swelling was reflected on an amelioration of the overall arthritic index in both antigens-treated groups versus the untreated arthritic rats with a maximum score improvement on the 19^th^ day with ATSA and on the 25^th^ day with ASMA treatment (Fig 3d). However, it reached statistical significant values throughout the last 2 weeks only with the ASMA-treated rats (*p* = 0.001).

### Gait score

The untreated arthritic rats showed a prominent gait impairment as evident by the significant increase in gait score versus the normal control rats (*p* = 0.000). A reduction of the gait score was observed in both antigens-treated arthritic groups though it was significant only in the ASMA-treated rats when compared with the untreated arthritis group (*p* = 0.002). Fig 3e

### Body weight

In the first week following induction of arthritis, all CFA-injected rats showed a significant decrease in the body weight relative to the base line weight as detected on day seven (*p* = 0.0000). Some weight gain was then observed in all rats after 2 weeks. With the exception of a decline in the body weight that was observed on the third week with ATSA treatment, both antigens-treated rats continued to show a significant increase in the body weight thereafter (*p*<0.05), while progressive weight loss was observed in the untreated-arthritic group till the end of the study. Fig 3f

### Inflammatory cytokines (IL-17, IL-10 and IFN-γ)

**Fig 4:**
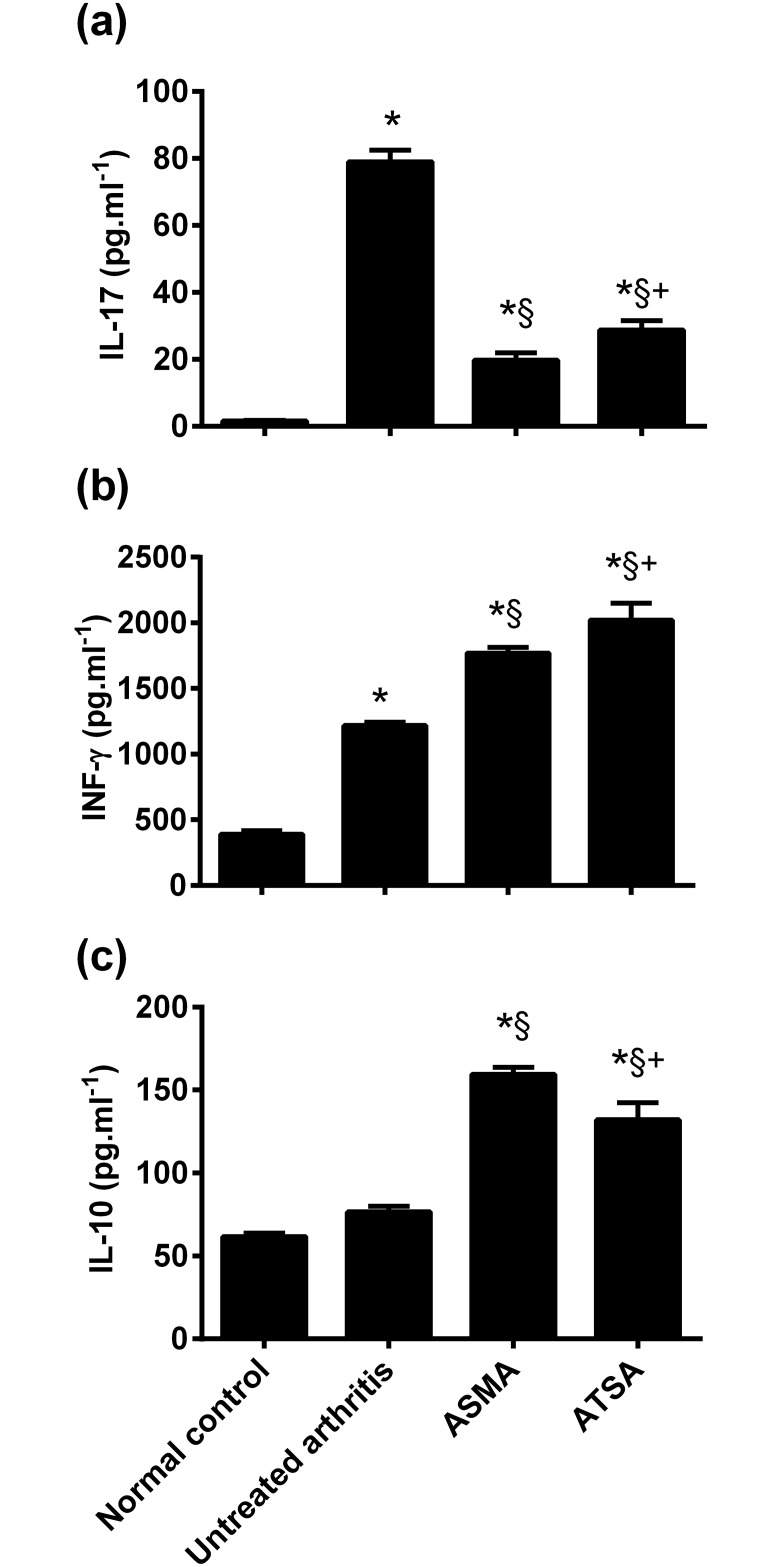
Effect of treatment with ASMA or ATSA on CFA-induced changes in inflammatory cytokines as *(a)*, IL-17, *(b)*, INF-γ and *(c)*, IL-10. Data are expressed as means ± S.E.M. *p < 0.05 versus normal control group, **§***p*< 0.05 versus untreated arthritis group and +*p* <0.05 versus ASMA-treated arthritis group. CFA, complete Freund’s adjuvant; ASMA, autoclaved *Schistosoma mansoni* antigen and ATSA, autoclaved *Trichinella spiralis* antigen.

A significant increase in the serum levels of IL-17 and IFN-γ was observed in the untreated arthritis group versus the normal control rats (*p* = 0.0000 for both cytokines), while the observed increase in IL-10 was found to be non-significant (*p* = 0.08). Concurrent treatment with either ASMA or ASTA antigens was associated with a significant decrease in IL-17 when compared with untreated arthritic group (*p* = 0.0000 for both antigens) with a significant lower level in the ASMA versus the ATSA-treated rats (*p* = 0.02). On the other hand, levels of both IL-10 and IFN-γ were found to be significantly elevated in both ASMA and ASTA-treated versus the untreated arthritis rats (*p* = 0.0000 for both antigens and both cytokines). A significant difference was also observed between the two antigens-treated groups (*p* = 0.003 and 0.018 for Il-10 and IFN-γ, respectively). Fig 4

### Histopathological joint inflammatory changes

**Fig 5:**
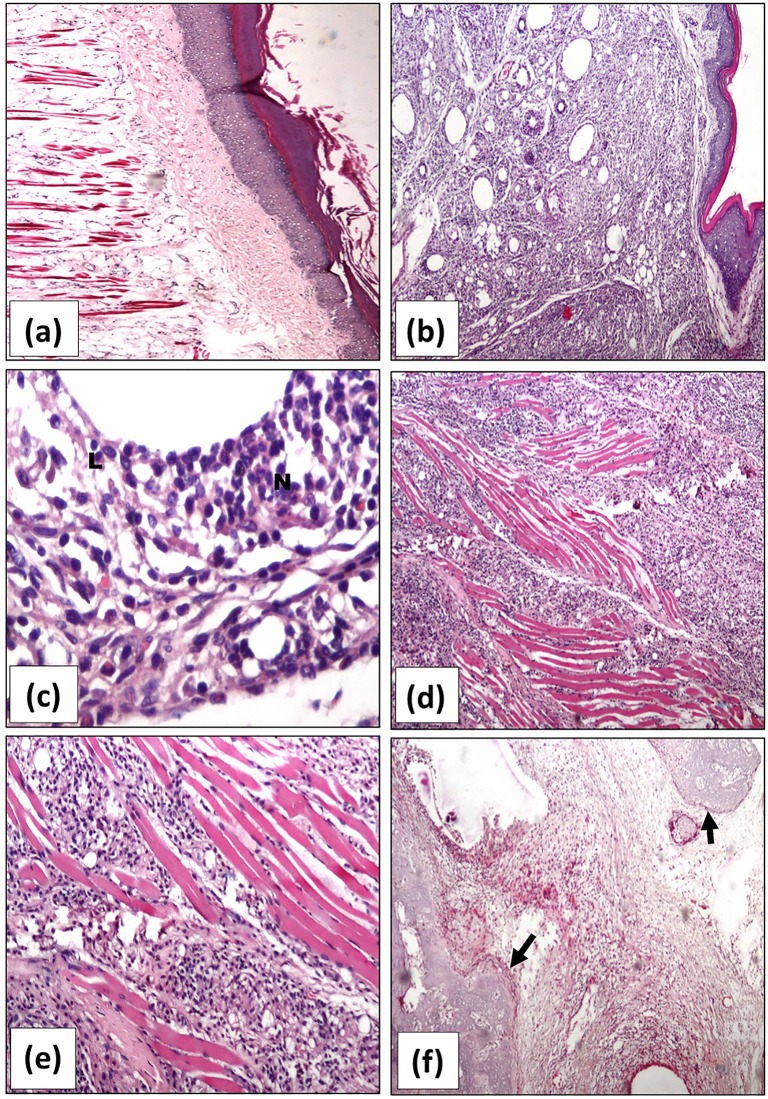
Representative photomicrographs of H&E stained sections of rats’ right hind paws. *(****a)***, joint tissue section of normal control rat showing normal articular structure without any inflammatory activity. *(****b-f)***, joint tissue sections of untreated arthritis group showing severe arthritis with intense inflammation in the subcutaneous tissue *(****b)*** with a high power view showing inflammatory cells mainly neutrophils (N) and lymphocytes (L) (x400) in ***(c)*.** The inflammation extends in between the muscle tissue in ***(d)*** and ***(e)*** (x200 and 400, respectively). Severe inflammatory reaction (↑) in the joint space with bone destruction is demonstrated in ***(f)*** (x 200).

Histopathological examination of H&E stained joint tissue sections obtained from normal control rats showed normal looking synovial membrane, no edema and no inflammatory cells (Fig 5a). On the other hand, cases of the untreated arthritis group showed severe acute inflammatory reaction with marked edema of the subcutaneous tissue, dense infiltrate of acute inflammatory cells mainly polymorphs, lymphocytes, scattered giant cells (Fig 5b and 5c). The diffuse inflammatory reaction infiltrates the subcutaneous tissue, muscles (Fig 5d and 5e), joint spaces, synovial membrane, even the bone trabeculae (Fig 5f). Inflammation was more remarkable on the right hind paw than that observed in sections of the left or the forepaw.

**Fig 6:**
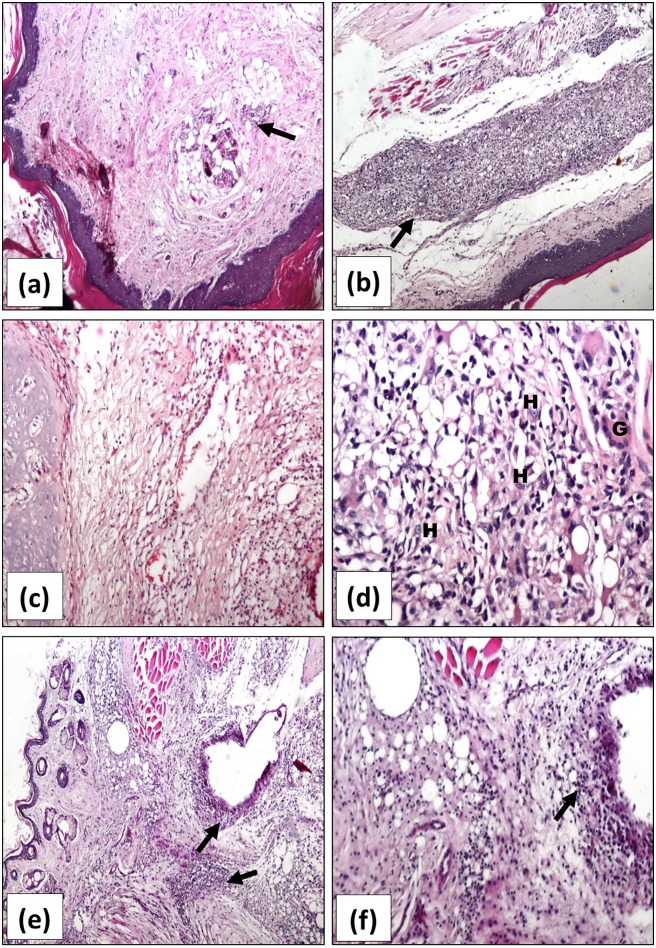
**Representative photomicrographs of H&E stained sections of rats’ right hind paws** of ASMA-treated in *(****a-d)*** and ATSA-treated arthritis groups in *(****e)*** and *(****f)***, showing reduced joint inflammation with mild in *(****a****)* and localized moderate inflammation (↑) in *(****b)*** and *(****c)*** (x200). Mixed inflammatory infiltrates with giant cells (G) and histiocytes (H) are evident with high power x400 in *(****d)*.** *(****e)*** and *(****f****)* show moderate inflammatory infiltration and edema of joints in ATSA-treated arthritis group (x200 and x400, respectively). ASMA; Autoclaved *Schistosoma Mansoni* antigen, ATSA; Autoclaved *Trichinella Spiralis* antigen.

All the examined sections obtained from the ASMA-treated arthritis rats showed remarkable regression of inflammation and moderate infiltrates of inflammatory cells with more predominance of chronic inflammatory cells; lymphocyte, histiocytes scattered granulomata with epithelioid cells, and giant cells (Fig 6a–6d). It is to be noted that inflammation is limited to the subcutaneous tissue. No inflammation is seen in muscles or synovial membranes. Similarly, relative regression of inflammatory reaction with moderate arthritis and residual few polymorphs was observed in tissue sections of the ATSA-treated arthritis group (Fig 6e and 6f).

**Fig 7:**
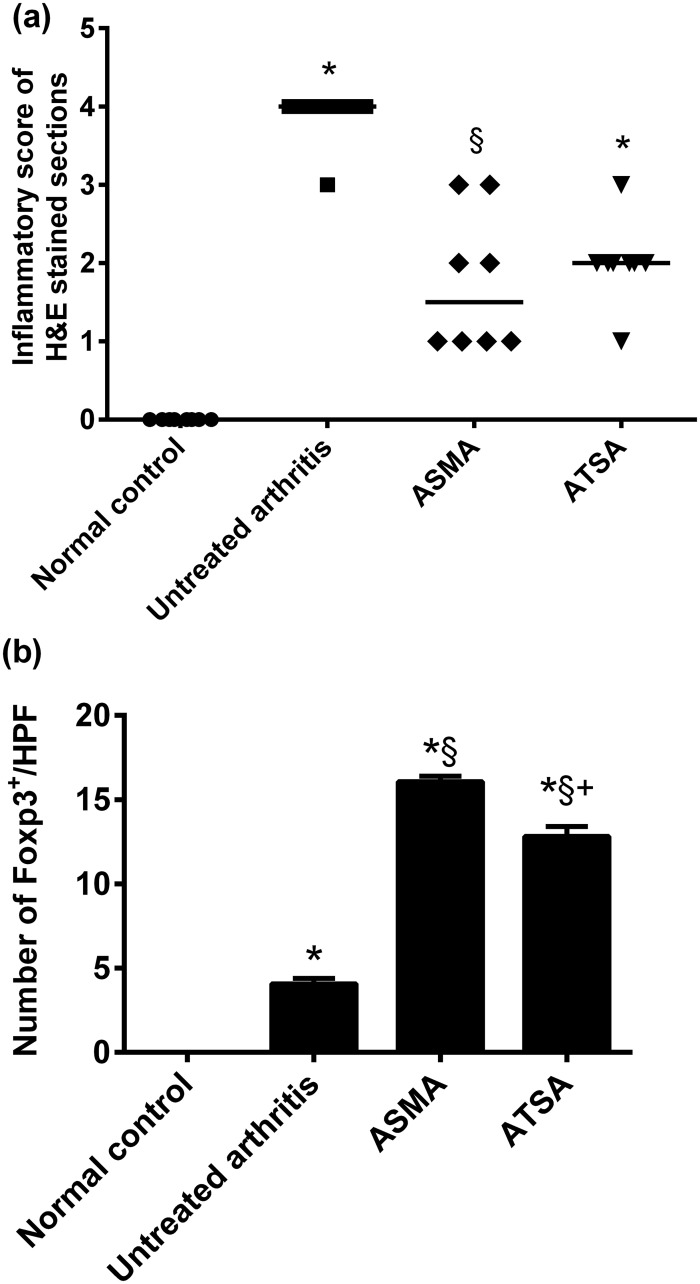
***(a)*,** scatter plot of semi-quantitative inflammatory score of H&E stained tissue sections of rats’ right hind paws. ***(b)***, number of Foxp3^+^ Treg cells in immunohistochemically stained tissue sections of rats’ right hind paws. Data are expressed as medians in ***(a)*** and as means ± S.E.M in ***(b)***. *p< 0.05 versus normal control group, **§***p*< 0.05 versus untreated arthritis group and **+***p*<0.05 versus ASMA-treated arthritis group. ASMA, autoclaved *Schistosoma mansoni* antigen and ATSA, autoclaved *Trichinella spiralis* antigen.

Statistical analysis of the semi-quantitative graded inflammatory score confirmed the significant attenuation of joint inflammation by ASMA (*p* = 0.037), while a non-significant decrease was observed with ATSA (*p* = 0.1) in comparison to the vehicle-treated arthritis group (Fig 7a).

### Number of Foxp^3+^ cells in the affected joints

**Fig 8:**
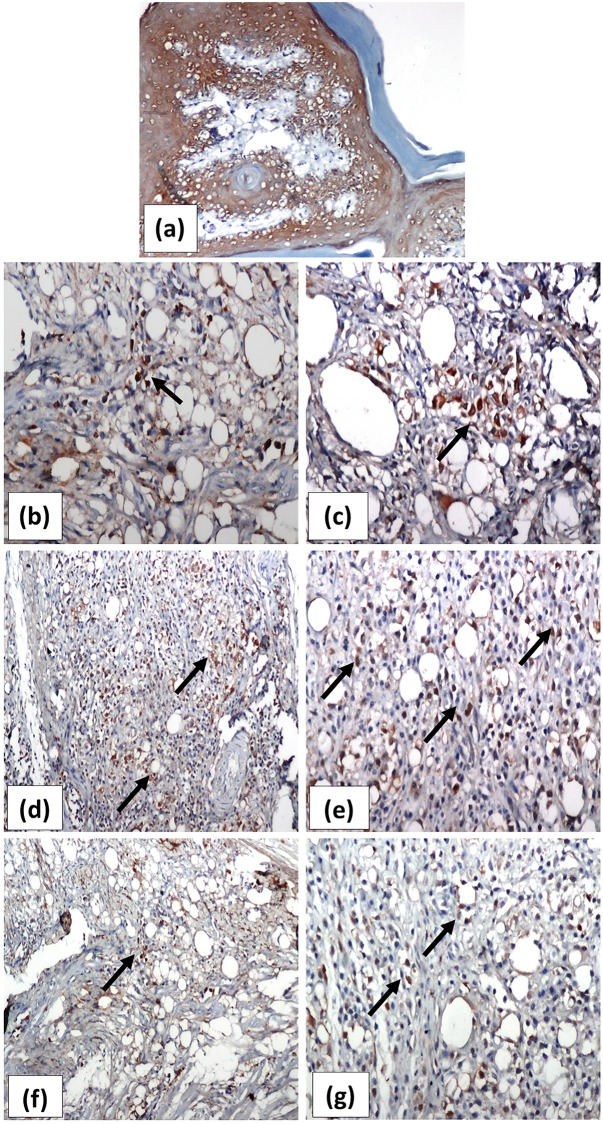
**Representative photomicrographs immunohistochemically stained sections of rats’ right hind paws with the anti-foxp3 antibody** showing normal control rat ‘s paw in ***(a)*** with no inflammatory infiltrate and absent foxp3^+^ cells (x100). Scattered foxp3^+^ cells (↑) with brown stained nuclei of untreated arthritis group are shown in *(****b)*** and *(****c)*** (x200 and x400, respectively). *(****d)*** and *(****e)*** show marked increase in the foxp3^+^ cells in joints of ASMA-treated arthritis rat (x200 and x400, respectively). Moderate increase in the number of fox p3^+^ cells after treatment with ATSA is shown in ***f*** (x200) with its high power magnification in *(****g)*** (x400). ASMA; Autoclaved *Schistosoma Mansoni* antigen, ATSA; Autoclaved *Trichinella Spiralis* antigen.

Examination of immunohistochemical stained joint sections of normal control rat showed no foxp3 positive cells, while few scattered cells ranging from 1–6 cells/ HPF (x400) was observed in sections of the untreated arthritis group. Treatment with either ASMA or ATSA was associated with a significant increase in the number of Foxp^3+^ cells versus the untreated arthritic group (*p* = 0.0000 for both antigens) ranging from 7-23/HPF and 8-20/HPF for ASMA and ATSA-treated groups, respectively with a significant difference versus each other (*p* = 0.0000). Figs 7b and 8

## Discussion

The inverse relationship observed long time ago between the prevalence of helminthic infections and that of RA and other autoimmune diseases raises much interest in the concept of helminthic therapy [35, 36]. Studies entailing the deliberate infection with live helminth parasites aiming to damp the abnormal immune response have yielded promising results [37]. Nevertheless, the detrimental effects of helminthic infection induced pathology remains a major concern. Therefore, the use of helminthic derived antigens that may replicate the benefits of helminthic infection on immune modulation, without the hazard of incurring parasitic disease, is now gaining more ground [38]. Herein, we investigated the effect of antigens derived from *S*. *mansoni* or *T*. *spiralis* in rat model of RA, a debilitating autoimmune disease that is not uncommonly refractory to existing conventional and biologic therapies.

The major findings of this study support a protective effect of both antigens against progression of arthritis both clinically and histopathologically. This was associated with general improvement in the overall health state as depicted from regaining weight. Mechanistic analysis revealed favourable modulation of key cytokines which may stem from an underlying upregulation of Foxp3^+^Treg cells.

Despite the multitude of studies proving the immune modulatory role of *T*. *spiralis* in autoimmune and allergic diseases using either crude muscle larval antigens, excretory products or infection [7, 39] its role in RA has not been addressed. To the best of our knowledge, this is also the first study demonstrating the ameliorative effect of ASMA on the clinical signs and structural derangements in AA. However, several proof-of-principal studies on *Schistosoma* species have been described earlier. Osada *et al*. demonstrated that prophylactic infection with *S mansoni* attenuated collagen II-induced arthritis in mice [14]. Similar results were also reported with *S*. *japonicum* infection [40]. Meanwhile, Sun *et al*. demonstrated a therapeutic potential of an *S*. *japonicum*-derived recombinant protein molecule (rSj16), in AA in rats [41].

In these studies, it was concluded that the generated immune response against infection stands behind the modulatory effect of helminths on host immune response to collagen arthritis. A well-recognized feature was that the Th2-polarization evoked in response to helminth infection would in theory have the ability to suppress pro-inflammatory Th1 responses that are mainly involved in generating dysregulated immune reaction in RA [10]. Hence, increased protective Th2 cytokines (e.g. IL-4) and suppression of the pathogenic Th1 cytokines (TNF-α and IFN-γ) have been proposed to explain the anti-arthritic effect of helminths. Our finding showed increased level of IFN-γ in AA but counterintuitively, further increase was detected with ASMA and ATSA treatment, in association with clinical and microscopic improvements. Indeed, the role of IFN-γ in experimental immune diseases is controversial, and in some models, a protective rather than a disease promoting effect is dominating [42]. Interestingly, Matthys *et al*. proposed that IFN-γ may induce both effects according to its source. In other words, the disease promoting effect of locally produced IFN-γ in affected tissues, might be overruled by the protective effect of the systemically produced IFN-γ [43]. This was attributed to its anti-proliferative action on certain mononuclear cell populations or to induction of suppressor cells. Furthermore, they demonstrated that the protective effect of IFN-γ is dependent on the presence of mycobacterial component in the CFA which opens a pathway by which endogenous IFN-γ exerts a protective effect that supersedes its otherwise disease-promoting effect [43]. Herein, and given this specific action, it can be assumed that the observed increase of IFN-γ in the untreated arthritic rats was an inadequate attempt by the immune system to provide protection. Higher levels of IFN-γ attained with ASMA and ASTA treatment may thus contribute to their anti-arthritic effect in this model of AA in which Mycobacteria constitute the main antigenic component. In line, *T*. *spiralis* antigens of different stages were reported to induce IFN-γ comparable to that induced by lipopolysaccharide (LPS) as a part of a mixed Th1/Th2 cytokine profile [44]. Regarding *S*. *mansoni*, the current increase in IFN-γ by ASMA in this AA rat model can be of more clinical value in light of the old but unique study of Cêtre et al. [45]. They demonstrated that despite the fact that rats are semi-permissive host, their humoral response to *S*. *mansoni* infection (which is cytokine dependent) is similar to human. In their study, a significant increase in IFN-γ was observed on day 21 post infection in response to soluble cercarial antigen. Though Sun *et al*. worked on the same model of AA, they reported a pathogenic role of IFN-γ that was attenuated with *S japonicum* derived protein [41]. However, the rSj16 induced changes in IFN-γ production were detected *in-vitro* in response to LPS exposure rather than detection in the serum in the AA model *in-vivo*.

In AA, the joint swelling, lymphocyte infiltration, and cartilage degradation are shared features with human RA. Moreover, IL-17 is an important pro-inflammatory cytokine expressed in the joints of RA patients as well as during the early stages of inflammation in AA and remains thereafter [1, 46].

Among other cytokines, IL-17 is involved in the pathology of both RA and AA. It increases inflammatory cellular infiltration into the synovium, promotes cartilage degradation and synergizes with tumour necrosis factor–α (TNF-α) for inducing bone resorption [47]. Furthermore, its blockade by anti-IL-17 antibody has been reported to ameliorate AA in rats [48]. One of our important findings is that treatment with either ASMA or ATSA was associated with marked attenuation of serum IL-17 with more prominent effect in the ASMA-treated rats. The inhibitory effects of helminthic antigens on IL-17 adds more insight into their anti-arthritic mechanisms. This IL-17 is mainly secreted by the CD4+ Th17 cells, a distinct lineage from CD4+ Th1 cells, which are traditionally claimed to be involved in RA. The recognition of the role of IL-17/IL-23 axis and other cytokines, revolutionized studies on the immunopathogenesis of RA and changed the classical concepts of the CD4+ Th1/Th2 paradigm as the main source of cytokine-mediated events during the course of arthritis [49]. These studies revealed also that IFN-γ inhibits the activity of IL-17 [50, 51], a notion that further supports our finding on the protective role of IFN-γ in autoimmune arthritis and that its upregulation by helminthic antigens mediate, at least in part, their anti-arthritic activity.

The activity of the pathogenic effector T cells like Th1/Th-17 cells can be dampened by a variety of regulatory T cells, whose suppressive activity require the expression of the transcription factor Foxp3. These cells are either generated in the thymus as natural T regulatory (nTreg) cells or can be induced form naïve T cells in the periphery (iTreg) in response to environmental antigens. Suppressive functions of Treg on effector T cells may occur either directly via cell-to-cell contact or via their secreted cytokines, TGF-β and IL-10 [52]. The role of Treg cells in autoimmune diseases and especially in RA has been the focus of interest in the last years [53, 54]. Though not fully elucidated, changes in these cells can be linked to the degree of inflammation and thus remission and relapse in RA. Supporting this concept, a decrease in the percentage of peripheral blood Foxp3^+^ Treg cells was reported in RA patients, and this decrease correlated with the severity of the disease [55]. Counterintuitively, an increase in the number of Treg cells was detected in joints and peripheral blood of RA patients in another study. However, the cells in joint showed loss of their suppressive function and expression of Foxp3 and exhibited a change toward a pathogenic IL-!7 producing cells phenotype [56]. Among the raised hypotheses is that Treg cells function are deficient in RA, whereas Treg counts may vary and thus Treg cell expansion or transfer may represent a successful approach for the treatment of RA [57].

Herein, immunohistochemical staining of joint tissue sections identified the existence of some Foxp3^+^ cells within the inflammatory infiltrate in untreated arthritic rats. Likewise, increased level of its suppressive cytokine, IL-10 was also detected in serum of these rats. This again may represent an inadequate trial of the immune system to circumscribe an excessive immune reaction in response to the CFA. Augmentation of both the number and function of Foxp3^+^ was clearly identified in each of ASMA and ATSA-treated arthritic groups and, this was associated with clinical improvement and alleviation of inflammatory reaction on histopathological examination. In support, infection with *T*. *spiralis* is reported to be accompanied by the accumulation of FoxP3+ Treg cells in the infected muscles during the chronic phase of infection [58]. Likewise, infection with *S*. *japonicum* stimulates the production of foxp3+Treg cells in humans [59]. Attenuation of pro-inflammatory cytokines as IL-17 is documented to be negatively correlated with expansion of Treg cells in many studies [60,61]. Of interest, the suppressive activity of Treg cells and its cytokine IL-10 is not only limited to damping over active pro-inflammatory Th1/Th17 subset of effector cells, but also extends to serve as inhibitor of osteoclastogenesis and bone damage [49]. Individual cases of severe bone damage and spontaneous amputation has been observed in untreated arthritic rats. On the contrary, no signs of bone damage were observed in either the ASMA or ATSA treated rats.

**In conclusion,** to the best of our knowledge, this study demonstrated for the first time the protective effect of *S*. *mansoni* derived antigen, rather than infection, in AA. It also sheds the first light on the anti-arthritic potential of *T*. *spiralis*. By upregulation of foxp3^+^ Treg cells, and augmentation of its suppressive activity via increased IL-10, both antigens dampened the production of the key pathogenic cytokine IL-17. This work also provides more insight into the paradoxical role of IFN-γ in this model of autoimmune arthritis. The use of autoclaved parasitic antigens excludes the deleterious effects of imposing helminthic infection using live parasites. However, further in depth studies are needed to characterize the exact molecule(s) responsible for immune modulation.

## References

[1] Bevaart L, Vervoordeldonk MJ, Tak PP. Evaluation of therapeutic targets in animal models of arthritis: how does it relate to rheumatoid arthritis. Arthritis Rheum. 2010; 62 (8): 2192–205. 10.1002/art.27503 20506322

[2] Weinstock JV, Summers RW, Elliott DE, Qadir K, Urban JF Jr, Thompson R. The possible link between de-worming and the emergence of immunological disease. J Lab Clin Med. 2002 6;139(6):334–8. 12066130 10.1067/mlc.2002.124343

[3] Okada H, Kuhn C, Feillet H, Bach JF. The 'hygiene hypothesis' for autoimmune and allergic diseases: an update. Clin Exp Immunol. 2010 4;160(1):1–9. 10.1111/j.1365-2249.2010.04139.x 20415844 PMC2841828

[4] Ngoi SM, Sylvester FA, Vella AT. The role of microbial byproducts in protection against immunological disorders and the hygiene hypothesis. Discov Med. 2011 11;12(66):405–12. 22127111

[5] Kondrashova A, Seiskari T, Ilonen J, Knip M, Hyöty H. The 'Hygiene hypothesis' and the sharp gradient in the incidence of autoimmune and allergic diseases between Russian Karelia and Finland. APMIS. 2013 6;121(6):478–93. 10.1111/apm.12023 23127244

[6] Bach JF. The effect of infections on susceptibility to autoimmune and allergic diseases. N Engl J Med.2002; 347:911–20. 10.1056/NEJMra020100 12239261

[7] Ashour DS. *Trichinella spiralis* immunomodulation an interactive multifactorial process. Expert Rev Clin Immunol. 2013; 9 (7): 669–75. 10.1586/1744666X.2013.811187 23899237

[8] Zaccone P, Hall SW. Helminth infection and type 1 diabetes. Rev Diabet Stud. 2012; 9(4):272–86. 10.1900/RDS.2012.9.272 23804266 PMC3740696

[9] Zaccone P, Fehervari Z, Phillips JM, Dunne DW, Cooke A. Parasitic worms and inflammatory diseases. Parasite Immunol. 2006; 28(10):515–23. 10.1111/j.1365-3024.2006.00879.x 16965287 PMC1618732

[10] Matisz CE, McDougall JJ, Sharkey KA, McKay DM. Helminth parasites and the modulation of joint inflammation. J Parasitol Res. 2011; 2011:942616 10.1155/2011/942616 21584243 PMC3092582

[11] El-Malky M, Nabih N, Heder M, Saudy N, EL-Mahdy M. Helminth infections: therapeutic potential in autoimmune disorders. Parasit Immunol. 2011; 33: 589–93. 10.1111/j.1365-3024.2011.01324.x 21797885

[12] Wang S, Xie Y, Yang X, Wang X, Yan K, Zhong Z, et al Therapeutic potential of recombinant cystatin from Schistosoma japonicum in TNBS-induced experimental colitis of mice. Parasit Vectors. 2016 1 4;9:6 10.1186/s13071-015-1288-1 26728323 PMC4700642

[13] Sega Y, Versini M, Shoenfeld Y. Of worms and men—Administration of helminth products as an innovative approach to treatment ofautoimmune diseases. Harefuah. 2015 7;154(7):428–31, 470, 469 26380461

[14] Osada Y, Shimizu S, Kumagai T, Yamada S, Kanazawa T. *Schistosoma mansoni* infection reduces severity of collagen induced arthritis via down-regulation of pro-inflammatory mediators. Int J Parasitol. 2009; 39 (4): 457–64. 10.1016/j.ijpara.2008.08.007 18835272

[15] Saunders KA, Raine T, Cooke A, Lawrence CE. Inhibition of autoimmune type 1 diabetes by gastrointestinal helminth infection. Infect Immun. 2007; 75(1): 397–407. 10.1128/IAI.00664-06 17043101 PMC1828378

[16] Park HK, Cho MK, Choi SH, Kim YS, Yu HS. *Trichinella spiralis*: infection reduces airway allergic inflammation in mice. Exp Parasitol. 2011; 127(2), 539–44. 10.1016/j.exppara.2010.10.004 21044628

[17] Ashour DS, Othman AA, Shareef MM, Gaballah HH, Mayah WW. Interactions between *Trichinella spiralis* infection and induced colitis in mice. J Helminthol. 2014; 88 (2):210–18. 10.1017/S0022149X13000059 23402295

[18] Gruden-Movsesijan A, Ilic N, Mostarica-Stojkovic M, Stosic-Grujicic S, Milic M, Sofronic-Milosavljevic L. Mechanisms of modulation of experimental autoimmune encephalomyelitis by chronic *Trichinella spiralis* infection in Dark Agouti rats. Parasite Immunol. 2010; 32(6): 450–59. 10.1111/j.1365-3024.2010.01207.x 20500676

[19] Bendele A. Animal models of rheumatoid arthritis. J Musculoskel Neuron Interact. 2001; 1 (4):377–85. 15758488

[20] McGrath JC, Lilley E. Implementing guidelines on reporting research using animals (ARRIVE etc.): new requirements for publication in BJP. Br J Pharmacol. 2015; 172: 3189–93. 10.1111/bph.12955 25964986 PMC4500358

[21] Pellegrino J, Katz N. Experimental chemotherapy of *Schistosomiasis mansoni*. Adv Parasitol. 1968; 6: 233–90. 4978052 10.1016/s0065-308x(08)60475-3

[22] Pica-Mattoccia L, Cioli D. Sex- and stage-related sensitivity of *Schistosoma mansoni* to *in vivo* and *in vitro* praziquantel treatment. Int J Parasitol. 2004; 34: 527–33. 10.1016/j.ijpara.2003.12.003 15013742

[23] El Aswad Bel D, Doenhoff MJ, El Hadidi AS, Schwaeble WJ, Lynch NJ. Use of recombinant calreticulin and cercarial transformation fluid (CTF) in the serodiagnosis of *Schistosoma mansoni*. Immunology. 2011; 216 (3): 379–85. 10.1016/j.imbio.2010.06.014 20691496

[24] Wasson DL, Dougherky DA, Dick TA. *T*. *Spiralis* induced by different *Trichinella* isolates. J Parasitol. 1988; 74 (2): 283–87. 3357119

[25] Eissa MM, El-Azzouni MZ, Mady RF, Fathy FM, Baddour NM. Initial characterization of an autoclaved *Toxoplasma* vaccine in mice. Exp Parasitol. 2012; 131: 310–16. 10.1016/j.exppara.2012.05.001 22595548

[26] Lowry OH, Rosebrough NS, Fan AL, Randall RS. Protein measurement with the Folin Phenol Reagent. J Biol Chem. 1951; 193:265–75. 14907713

[27] Darwish SF, El-Bakly WM, Arafa HM, El-Demerdash E. Targeting TNF-α and NFκB activation by bee venom: role in suppressing adjuvant induced arthritis and methotrexate hepatotoxicity in rats. PLoS One. 2013; 8(11): e79284 10.1371/journal.pone.0079284 24278124 PMC3835890

[28] Eissa MM, Allam SR, El-Azzouni MZ, Maged HR, Dessouky IS. Further studies on Autoclaved cercarial vaccine against schistosomiasis: safety, longevity and stability. J Egypt Soc Parasitol. 2003; 33 (2): 541–60. 14964666

[29] Darani HY, Curtis RH, McNeice C, Price HP, Sayers JR, Doenhoff MJ. *Schistosoma mansoni*: anomalous immunogenic properties of a 27 kDa larval serine protease associated with protective immunity. Parasitology. 1997; 115(3): 237–47. 9300461 10.1017/s0031182097001303

[30] Wang XL, Fu BQ, Yang SJ, Wu XP, Cui GZ, Liu MF, et al *Trichinella spiralis*—A potential anti-tumor agent. Vet Parasitol. 2009; 159: 249–52. 10.1016/j.vetpar.2008.10.052 19041180

[31] Hawkins P, Armstrong R, Boden T, Garside P, Knight K, Lilley E, et al Applying refinement to the use of mice and rats in rheumatoid arthritis research. Inflammopharmacology. 2015 (4):131–50. 10.1007/s10787-015-0241-4 26168847 PMC4508365

[32] Dief AE, Mostafa DK, Sharara GM, Zeitoun TH. Hydrogen sulfide releasing naproxen offers better anti-inflammatory and chondroprotective effect relative to naproxen in a rat model of zymosan induced arthritis. Eur Rev Med Pharmacol Sci. 2015; 19 (8):1537–46. 25967731

[33] Drury RAB, Wallington EA. Carleton Histological Technique, fifth ed Oxford University Press, Oxford, New York, Toronto 1980

[34] Rzepecka J, Pineda MA, Al-Riyami L, Rodgers DT, Huggan JK, Lumb FE, et al Prophylactic and therapeutic treatment with a synthetic analogue of a parasitic worm product prevent experimental arthritis and inhibits IL-1β production via NRF2-mediated counter-regulation of the inflammasome. J Autoimmun. 2015; 60:59–73. 10.1016/j.jaut.2015.04.005 25975491 PMC4459730

[35] Greenwood BM. Autoimmune disease and parasitic infections in Nigerians. Lancet. 1986; 2:380–2. 4174413 10.1016/s0140-6736(68)90595-3

[36] Finlay CM, Stefanska AM, Walsh KP, Kelly PJ, Boon L, Lavelle EC, et al Helminth products protect against autoimmunity via innate type 2 cytokines IL-5 and IL-33, which promote eosinophilia. J Immunol. 2016; 196 (2): 703–14. 10.4049/jimmunol.1501820. Epub 2015 Dec 16. 26673140

[37] Fleming JO, Weinstock JV. Clinical trials of helminth therapy in autoimmune diseases: rationale and findings. Parasite Immunol.2015; 37: 277–92. 10.1111/pim.12175 25600983

[38] Maizels RM. Parasitic helminth infections and the control of human allergic and autoimmune disorders Clin Microbiol Infect. 2016; 22 (6):481–6. 10.1016/j.cmi.2016.04.024 27172808

[39] Yang X, Yang Y, Wang Y, Zhan B, Gu Y, Cheng Y, et al Excretory/secretory products from *Trichinella spiralis* adult worms ameliorate DSS-induced colitis in mice. PLoS One. 2014;9 (5): e96454 10.1371/journal.pone.0096454. eCollection 2014. 24788117 PMC4008629

[40] He Y, Li J, Zhuang W, Yin L, Chen C, Li J, et al “The inhibitory effect against collagen-induced arthritis by *Schistosoma japonicum* infection is infection stage-dependent,” BMC Immunol. 2010; 11:128–33. 10.1186/1471-2172-11-28 PMC2891676 20537152

[41] Sun X, Liu YH, Lv ZY, Yang LL, Hu SM, Zheng HQ, et al rSj16, a recombinant protein of *Schistosoma japonicum* derived molecule, reduces severity of complete Freund’s adjuvant-induced adjuvant arthritis in rats' model. Parasite Immunol. 2010; 32 (11–12):739–48. 10.1111/j.1365-3024.2010.01240.x 21039614

[42] Krakowski M, Owens T. Interferon-g confers resistance to experimental allergic encephalomyelitis. Eur J Immunol. 1996; 26:1641 10.1002/eji.1830260735 8766573

[43] Matthys P, Vermeire K, Mitera T, Heremans H, Huang S, Schols D, et al Enhanced autoimmune arthritis in IFN-gamma receptor-deficient mice is conditioned by mycobacteria in Freund's adjuvant and by increased expansion of Mac-1+ myeloid cells. J Immunol. 1999; 163 (6):3503–10. 10477624

[44] Ilic N, Worthington JJ, Gruden-Movsesijan A, Travis MA, Sofronic-Milosavljevic L, Grencis RK. *Trichinella spiralis* antigens prime mixed Th1/Th2 response but do not induce de novo generation of Foxp3+ T cells *in vitro*. Parasite Immunol. 2011; 33 (10):572–82. 10.1111/j.1365-3024.2011.01322.x 21793858 PMC3485669

[45] Cêtre C, Pierrot C, Cocude C, Lafitte S, Capron A, Capron M, et al (1999). Profiles of Th1 and Th2 cytokines after primary and secondary infection by *Schistosoma mansoni* in the semipermissive rat host. Infect Immun. 1999;67(6) 2713–19. 10338473 10.1128/iai.67.6.2713-2719.1999 PMC96574

[46] Wu H, Yan S, Chen J, Luo X, Li P, Jia X, et al JAK1-STAT3 blockade by JAK inhibitor SHR0302 attenuates inflammatory responses of adjuvant-induced arthritis rats and decreases Th17 and total B cells. Joint Bone Spine.2016; pii: S1297-319X (15) 00282–1. 10.1016/j.jbspin.2015.09.002 26832189

[47] Chabaud M, Lubberts E, Joosten L, van den Berg W, Miossec P. IL-17 derived from juxta-articular bone and synovium contributes to joint degradation in rheumatoid arthritis. Arthritis Res 2001; 3: 168–77. 10.1186/ar294 11299057 PMC30709

[48] Lubberts E, Koenders MI, Oppers-Walgreen B, Lubberts E, Koenders MI, Oppers-Walgreen B, et al Treatment with a neutralizing anti-murine interleukin-17 antibody after the onset of collagen-induced arthritis reduces joint inflammation, cartilage destruction, and bone erosion. Arthritis Rheum. 2004; 50:650–9. 10.1002/art.20001 14872510

[49] Moudgil KD, Kim P, Brahn E. Advances in rheumatoid arthritis animal models. Curr Rheumatol Rep. 2011; 13 (5):456–63. 10.1007/s11926-011-0200-z 21792748 PMC3786133

[50] Rajaiah R, Puttabyatappa M, Polumuri SK, Moudgil KD (2011). Interleukin-27 and Interferon-{gamma} are involved in regulation of autoimmune arthritis. J Biol Chem. 2011; 286(4):2817–25. 10.1074/jbc.M110.187013 21123181 PMC3024777

[51] Doodes PD, Cao Y, Hamel KM, Wang Y, Rodeghero RL, Mikecz K, et al IFN-gamma regulates the requirement for IL-17 in proteoglycan-induced arthritis. J Immunol. 2010; 184:1552–59. 10.4049/jimmunol.0902907 20028652 PMC2846113

[52] Mellor AL, Munn DH. Physiologic control of the functional status of FOXP3^+^ regulatory T cells. J Immunol. 2011; 186: 4535–40. 10.4049/jimmunol.1002937 21464094 PMC3808246

[53] Cooles FA, Isaacs JD, Anderson AE. Treg cells in rheumatoid arthritis: an update. Curr Rheumatol Rep. 2013 9;15(9):352 10.1007/s11926-013-0352-0 23888361

[54] Alunno A, Manetti M, Caterbi S, Ibba-Manneschi L, Bistoni O, Bartoloni E, et al Altered immunoregulation in rheumatoid arthritis: the role of T cells and proinflammatory Th17 cells and therapeutic implications. Mediators Inflamm. 2015; 2015:751793 10.1155/2015/751793 25918479 PMC4397010

[55] Sempere-Ortells JM, Pérez-García V, Marín-Alberca G, Peris-Pertusa A, Benito JM, Marco FM, et al Quantification and phenotype of regulatory T cells in rheumatoid arthritis according to disease activity score-28. Autoimmunity. 2009;42(8):636–45. 10.3109/08916930903061491 19886735

[56] Wang T, Sun X, Zhao J, Zhang J, Zhu H, Li C, et al Regulatory T cells in rheumatoid arthritis showed increased plasticity toward Th17 but retained suppressive function in peripheral blood. Ann Rheum Dis. 2015 6;74(6):1293–301. 10.1136/annrheumdis-2013-204228 24521740

[57] Boissier MC, Assier E, Biton J, Denys A, Falgarone G, Bessis N. Regulatory T cells (Treg) in rheumatoid arthritis. Joint Bone Spine. 2009 1;76(1):10–4. 10.1016/j.jbspin.2008.08.002 19028128

[58] Beiting DP, Gagliardo LF, Hesse M, Bliss SK, Meskill D, Appleton JA. Coordinated control of immunity to muscle stage *Trichinella spiralis* by IL-10, Regulatory T Cells, and TGF-b. J Immunol. 2007; 178: 1039–47. 17202367 10.4049/jimmunol.178.2.1039

[59] Romano A, Hou X, Sertorio M, Dessein H, Cabantous S, Oliveira P, et al FOXP3+ Regulatory T Cells in hepatic fibrosis and splenomegaly caused by *Schistosoma Japonicum*: The spleen may be a major source of Tregs in subjects with splenomegaly. PLoS Negl Trop Dis. 2016; 10 (2): e0004454 10.1371/journal.pntd.0004454 26731721 PMC4701139

[60] Weng L, Williams RO, Vieira PL, Screaton G, Feldmann M, Dazzi F (2010). The therapeutic activity of low-dose irradiation on experimental arthritis depends on the induction of endogenous regulatory T cell activity. Ann Rheum Dis. 2010; 69:1519–26. 10.1136/ard.2009.121111 20498214

[61] Lee SY, Min HK, Lee SH, Shin HJ, Lee WY, Cho YG, et al (2016). Park SH10IL- receptor antagonist (IL-1Ra)-Fc ameliorate autoimmune arthritis by regulation of the Th17 cells/Tregbalance and arthrogenic cytokine activation. Immunol Lett. 2016; 172:56–66. 26903194 10.1016/j.imlet.2016.02.011

